# Morphological, elemental, and boron isotopic insights into pathophysiology of diseased coral growth anomalies

**DOI:** 10.1038/s41598-020-65118-6

**Published:** 2020-05-19

**Authors:** Erik R. Andersson, Joseph A. Stewart, Thierry M. Work, Cheryl M. Woodley, Tracey B. Schock, Rusty D. Day

**Affiliations:** 1 Grice Marine Laboratory, Department of Biology, College of Charleston, 205 Fort Johnson Rd., Charleston, SC 29412 USA; 2 Marine Biochemical Sciences, Chemical Sciences Division, National Institute of Standards and Technology, Hollings Marine Laboratory, Charleston, SC 29412 USA; 3 School of Earth Sciences, University of Bristol, Queens Road, Bristol, BS8 1RJ UK; 4 U.S. Geological Survey National Wildlife Health Center, Honolulu Field Station, Honolulu, HI 96850 USA; 5 National Oceanic and Atmospheric Administration, National Ocean Service, National Centers for Coastal Ocean Sciences, Hollings Marine Laboratory, Charleston, SC 29412 USA; 6 Present Address: Marine Science and Nautical Training Academy (MANTA), 520 Folly Rd., Charleston, SC 29412 USA

**Keywords:** Ecology, Element cycles, Pathogenesis

## Abstract

Coral growth anomalies (GAs) are tumor-like lesions that are detrimental to colony fitness and are commonly associated with high human population density, yet little is known about the disease pathology or calcification behavior. SEM imagery, skeletal trace elements and boron isotopes (δ^11^B) have been combined as a novel approach to study coral disease. Low Mg/Ca, and high U/Ca, Mo/Ca, and V/Ca potentially suggest a decreased abundance of “centers of calcification” and nitrogen-fixation in GAs. Estimates of carbonate system parameters from δ^11^B and B/Ca measurements indicate reduced pH (−0.05 units) and [CO_3_^2−^] within GA calcifying fluid. We theorize GAs re-allocate resources away from internal pH upregulation to sustain elevated tissue growth, resulting in a porous and fragile skeleton. Our findings show that dystrophic calcification processes could explain structural differences seen in GA skeletons and highlight the use of skeletal geochemistry to shed light on disease pathophysiology in corals.

## Introduction

The high biodiversity found on coral reefs makes them one of the most ecologically and socio-economically important ecosystems in the world^1^. Yet, increasing stressors have led to a widespread decline in the overall reef health in recent decades^2^. Rising atmospheric carbon dioxide represents a global threat to coral reefs by increasing ocean temperatures and reducing ocean pH^3^, respectively causing wide-scale bleaching events^4^ and reducing calcification rates on coral reefs^5^. Localized stressors such as eutrophication, sedimentation, and chemical pollution also decrease health and resilience of coral reef organisms^6^. Disease outbreaks have the ability to decimate coral populations^7^ and are typically correlated with localized human impacts^8^. With mounting global and local stressors weakening coral resilience, disease has become more prevalent in recent decades and now represents a substantial threat to global coral reef health^9,10^. Most studies of coral diseases have focused primarily on field surveys, physiological measurements, and the culture/characterization of coral bacteria, yet crucial diagnostic aspects such as micro-scale pathology and skeletal chemistry have not been thoroughly investigated^11^. Furthermore, many diseases share similar visual presentation, but similar disease signs do not necessarily arise from a common causative agent, making exact causation unclear^12,13^.

Coral growth anomalies (GAs) affect multiple genera and are identified by localized abnormal skeletal growth resulting in a protuberant calcified mass on a coral colony^14^. GAs are generally characterized by rapid growth of less dense skeletal carbonate, with associated tissues having fewer polyps, fewer endosymbiotic dinoflagellates (family Symbiodiniaceae), and reduced reproductive potential^14,15^. Although these abnormal characteristics do not usually result in immediate mortality of an afflicted colony, the reduction in overall organism fitness makes GAs an ecological threat where prevalence is high^16^. GAs have been characterized in a variety of species using field surveys, histological, cellular, and molecular techniques^15,17–20^, yet the pathology of GAs remains unclear. Suggested causes include mutagenesis associated with exposure to UV radiation or pathogenic microorganisms^21,22^. To date, no conclusive evidence has been provided to support these hypotheses, however the linkage between human population size and the presence of GAs^23,24^ strongly indicates that local stressors influence GA occurrence.

Localized abnormal skeleton growth is a defining characteristic of coral GAs, therefore more detailed investigation of skeletal formation can be insightful towards understanding disease mechanisms. Corals calcify from an extracellular calcifying fluid (ECF) which is semi-isolated from surrounding seawater. Precipitation of aragonite depends on the saturation state (Ω = [Ca^2+^][CO_3_^2−^]/K) of the ECF, which can be modified to some degree by the organism. For example, pH and Ω can be raised within the ECF by Ca^2+^-ATPase enzymes that import Ca^2+^ in exchange for H^+^^25^. Trace metals are also incorporated into coral aragonite, either due to their role in calcification or as contaminants in the crystalline lattice.

Incorporation of trace elements generally scales with their abundance in ambient seawater, however, physiological influences on calcification (termed ‘vital effects’) can disrupt this relationship^26^. Comparing skeleton from corals grown under similar seawater chemistries should theoretically isolate these vital effects thus revealing direct and indirect biological controls on skeletal chemistry. Essential trace elements are necessary for important biological and metabolic processes, often acting as metal co-factors in a wide range of enzymes. These elements may therefore act as biomarkers for tracking activity of associated biochemical pathways as changes in enzyme (and metal co-factor) abundance alter ambient metal availability and thus elemental incorporation into the skeleton. By contrast, non-essential and toxic elements have no known biological function and may be harmful even at low concentrations and differences in these metals may indicate changes in their uptake or depuration rates. Some elements, such as boron or its isotopes (δ^11^B), can serve as useful markers of calcification. For instance, δ^11^B varies according to internal pH (pH_ECF_)^27–29^, and recent studies suggest that an all-important second carbonate system parameter can be calculated (thus allowing full carbonate system computation) using paired coral δ^11^B and B/Ca ratios to estimate internal carbonate ion concentration ([CO_3_^2−^]_ECF_)^30–32^. Collectively, the elemental and boron chemistries have the potential to reveal key differences in calcification processes, as well as differences in wider holobiont physiological and biochemical activity, between healthy and diseased corals.

The finger coral *Porites compressa* is an abundant and ecologically important reef-building coral that largely dominates the reefs of Kaneohe Bay in Oahu, Hawaii^33^. Kaneohe Bay is widely studied owing to its long history of anthropogenic impacts and is often considered to be an example of ecological resilience to environmental insult^33^. *P. compressa* are commonly afflicted by GAs in Kaneohe Bay^16,17^ and *P. compressa* GAs have been characterized in the past, including measurements of a limited suite of skeletal trace elements (Mg, Sr, Fe, Mn) with no differences detected^17^, however the study excluded many key essential and toxic metals that may provide useful information for our understanding of GA pathphysiology.

Here we present a detailed morphological description of *P. compressa* GAs collected from Kaneohe Bay (Coconut Island, Supplementary Fig. S1) and propose new species-specific nomenclature for different GA types in *P. compressa*. We supplement this with comprehensive skeletal chemical analyses of 20 trace metals and δ^11^B in paired GA and unaffected specimens of the same colony. These paired samples allow differences in trace elements and ECF carbonate system parameters to be assessed between GA and apparently healthy (unaffected) areas of a diseased colony exposed to identical environmental conditions. Such comparison gives fresh insight into pathophysiology of GAs that contributes to our overall knowledge of the physiological and ecological impacts of the disease.

## Results and Discussion

### Morphological characterization

**Figure 1:**
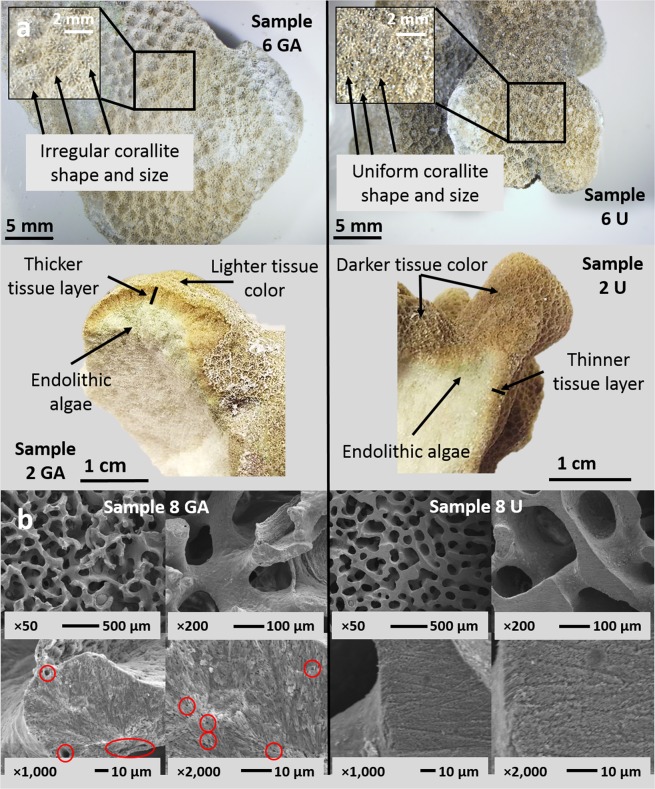
Images depicting *Porites compressa* morphological and skeletal characteristics. Digital (**a**) and scanning electron microscopy (**b**) images from representative growth anomaly (GA; left) and unaffected (U; right) *P. compressa* samples are shown. In panel B, note the thin walled trabeculae of GAs (lower left) which on cut surface reveal porous (red circles) matrix with haphazardly arranged aragonite crystals. Contrast that with dense homogenous normal skeleton (lower right) with orderly arrays of crystals on cut surface (lower right).

GA lesions range in size from 15.0 to 78.0 cm^3^ and all but three have distinct edges between the lesion and surrounding unaffected tissue (Table 1). When compared to unaffected tissue (n = 14) from the same colony, GA lesions (n = 14) are generally found to have (i) more irregularly shaped corallites with less defined theca and septa, (ii) lighter colored tissue that is typically indicative of lower density of symbiotic dinoflagellates (iii) endolithic algae that extended deeper into the coral skeleton (Fig. 1a) (iv) greater tissue depth, (v) greater corallite diameter, and (vi) lower corallite spatial density (Table 2, p < 0.05). These findings are generally in accordance with previous descriptions of GAs in *P. compressa*^16,17^. For example, the respective mean corallite diameters of 1.50 ± 0.05 mm and 1.36 ± 0.05 mm we measure for GA and unaffected samples are remarkably similar to those previously documented in *P. compressa* (1.48 ± 0.16 mm and 1.32 ± 0.14 mm respectively^17^).

**Table 1:** Growth anomaly (GA) lesion macro-morphology from each *Porites compressa* GA sample.

Sample ID	Edges	Shape	Relief	GA lesion size (cm^3^)	GA lesion form
1 GA	Indistinct	Irregular	Nodular	28.0	Form 2
2 GA	Distinct	Oblong	Umbonate to Bosselated	78.0	Form 1
3 GA	Distinct	Irregular	Nodular	32.0	Form 2
4 GA	Distinct	Oblong	Umbonate to Bosselated	26.2	Form 1
5 GA	Distinct	Oblong	Umbonate to Bosselated	23.6	Form 1
*6 GA	Unknown	Unknown	Unknown	Unknown	Unknown
7 GA	Distinct	Oblong	Umbonate to Bosselated	21.8	Form 1
8 GA	Indistinct	Oblong	Umbonate to Bosselated	54.0	Form 1
9 GA	Distinct	Oblong	Umbonate to Bosselated	15.0	Form 1
10 GA	Distinct	Oblong	Umbonate to Bosselated	60.0	Form 1
11 GA	Distinct	Oblong	Umbonate to Bosselated	35.0	Form 1
12 GA	Distinct	Oblong	Umbonate to Bosselated	17.5	Form 1
13 GA	Distinct	Oblong	Umbonate to Bosselated	31.5	Form 1
15 GA	Indistinct	Irregular	Nodular	61.8	Form 2

Distribution, location, edges, shape and relief described according to nomenclature of Work and Aeby^12^. *Descriptions could not be determined for sample 6 GA as it comprised only a portion of the entire lesion.

**Table 2:** Mean (±standard error of the mean) morphological and chemical measurements for growth anomaly (GA) and unaffected *Porites compressa* skeletons.

		Unaffected (n=14)	± SE	GA (n=14)	± SE	p-value (t-value; df)
**Morphology**						
Tissue Depth	mm	2.7	0.1	4.4	0.3	0.00077 (–5.85; 13)
Corallite Spatial Density	#/mm^2^	0.76	0.02	0.65	0.04	0.029 (2.92; 13)
Corallite Diameter	mm	1.36	0.05	1.50	0.05	0.013 (–3.43; 13)
**Trace Elements**						
Li/Ca	(µmol/mol)	6.91	0.11	6.79	0.06	0.39 (1.15; 13)
B/Ca	(µmol/mol)	552	7	553	7	0.99 (–0.10; 13)
Na/Ca	(mmol/mol)	22.27	0.30	22.16	0.23	0.44^b^
Mg/Ca	(mmol/mol)	5.14	0.08	4.70	0.07	0.00050 (6.55; 13)
Al/Ca	(µmol/mol)	5.98	0.85	7.59	1.02	0.071 (–2.33; 13)
V/Ca	(nmol/mol)	78	1	87	1	0.0014 (–5.07; 13)
Mn/Ca	(µmol/mol)	1.00	0.05	0.93	0.06	0.15 (1.80; 13)
Fe/Ca	(µmol/mol)	5.23	3.78	1.55	0.22	0.92^a^
Co/Ca	(µmol/mol)	0.462	0.007	0.468	0.005	0.85 ^a^
Ni/Ca	(µmol/mol)	5.55	0.02	5.52	0.02	0.20 (1.61; 13)
Cu/Ca	(µmol/mol)	0.266	0.022	0.265	0.027	0.99 (0.02; 13)
Zn/Ca	(µmol/mol)	25.2	4.6	25.2	4.4	0.99 (0.005; 13)
Rb/Ca	(nmol/mol)	10.79	0.17	10.81	0.15	0.99 (–0.06; 13)
Sr/Ca	(mmol/mol)	9.30	0.03	9.41	0.04	0.013 (–3.37; 13)
Mo/Ca	(nmol/mol)	7.7	0.2	8.6	0.2	0.0021 (–4.65; 13)
Sb/Ca	(nmol/mol)	6.91	0.17	7.56	0.14	0.0055 (–3.96; 13)
Ba/Ca	(µmol/mol)	47.37	10.18	34.61	7.29	0.85^b^
Nd/Ca	(nmol/mol)	1.60	0.16	2.39	0.23	0.0011^a^
Pb/Ca	(nmol/mol)	10.98	1.61	12.46	1.81	0.096 (–2.12; 13)
U/Ca	(µmol/mol)	1.060	0.032	1.219	0.044	0.0014 (–4.96; 13)
**Boron Systematics**						
δ^11^B	(‰)	24.13	0.20	23.36	0.23	0.044 (2.62; 13)
pH_ECF_		8.50	0.01	8.45	0.01	0.044 (2.63; 13)
[CO_3_^2−^]_ECF_	(µmol/kg)	971	15	901	13	0.0047 (4.18; 13)

Paired sample t-tests were primarily used to test for differences between groups. Where some samples were less than the limits of detection (^a^) or differences between pairs were not normally distributed (^b^) a Wilcoxon signed-rank test was alternatively used. For those elements with non-detects, a robust regression on order statistics was used to estimate group mean and standard error. Internal pH is calculated from δ^11^B using formula by Dickson^74^ and [CO_3_^2−^]_ECF_ is calculated from δ^11^B and B/Ca using method of DeCarlo *et al*.^32^. Benjamini-Hochberg false discovery rate^80^ was used to adjust p-values for multiple comparisons.

**Figure 2:**
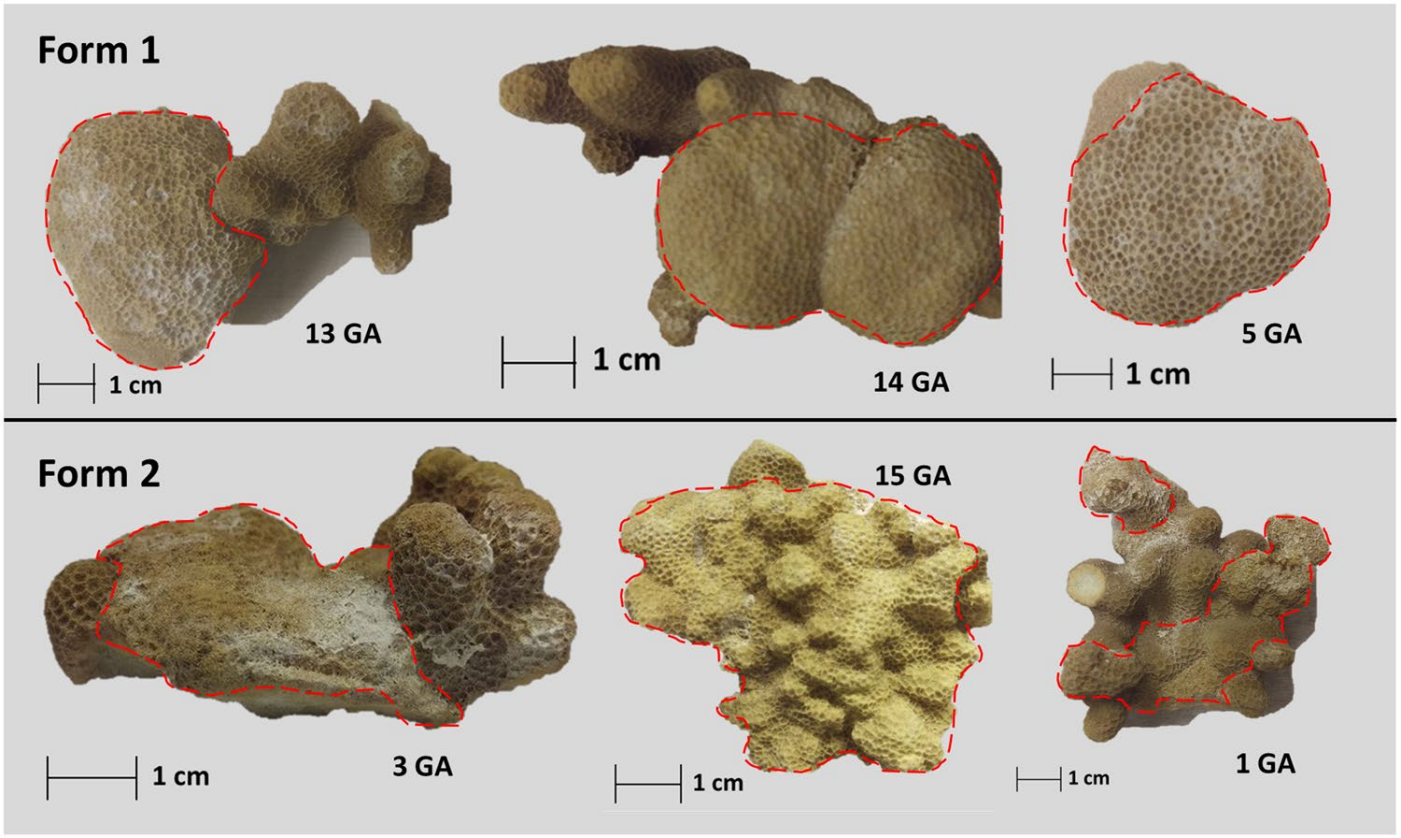
Examples the two described growth anomaly lesion macro-morphology forms. Form 1 with lesions distinguished by an oblong shape and umbonate to bosselated relief, and Form 2 with lesions distinguished by an irregular shape and nodular relief. Dashed red lines indicate growth anomaly (GA) lesion area.

We take categorization further and describe two distinct GA forms primarily based on differences in shape and relief of the lesions. We classify lesions with an oblong shape and umbonate to bosselated (bulbous) relief as “Form 1”, which are protuberant and bosselated similar to those previously described in *P. compressa*^16,17^. By contrast, “Form 2” lesions are shorter with a wider base, less protuberant and have a nodular surface, thus representing a heretofore undescribed morphology (Fig. 2, Supplementary Fig. S2).

**Figure 3:**
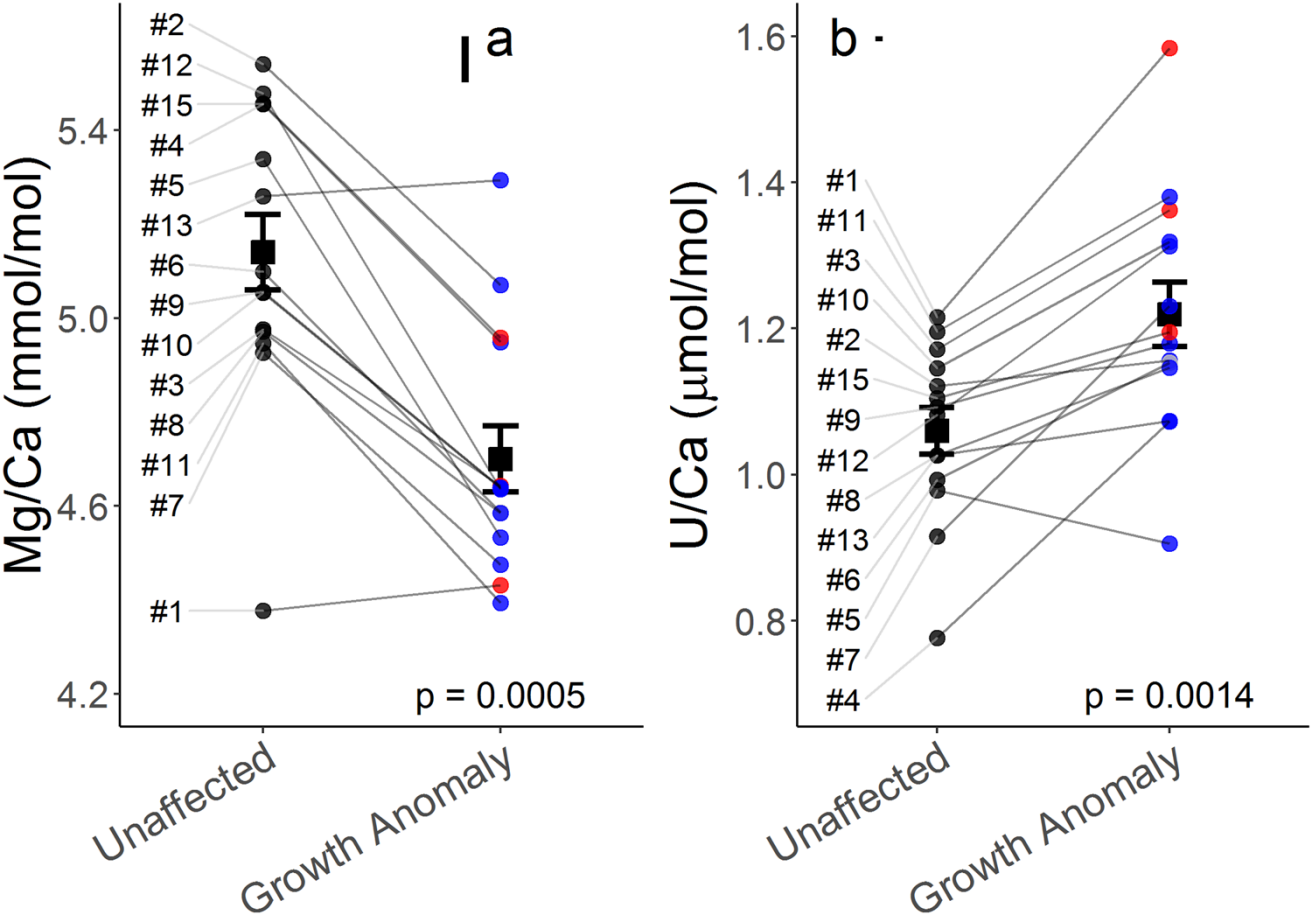
Skeletal measurements linked to chemistry in centers of calcification from unaffected and growth anomaly *Porites compressa*. Average and individual measurements of Mg/Ca (**a**) and U/Ca (**b**) ratios are shown for unaffected (n = 14) and growth anomaly (n = 14) samples. Black squares represent group mean (± standard error of the mean) and black (unaffected), blue (Form 1), red (Form 2) and grey (unclassified) circles indicate individual sample morphology. Sample numbers are indicated along the left margin and grey lines connect each paired set of unaffected and growth anomaly samples. Floating black bars indicate analytical uncertainty for Mg/Ca and U/Ca. Presented p-values result from paired sample t-tests.

**Figure 4:**
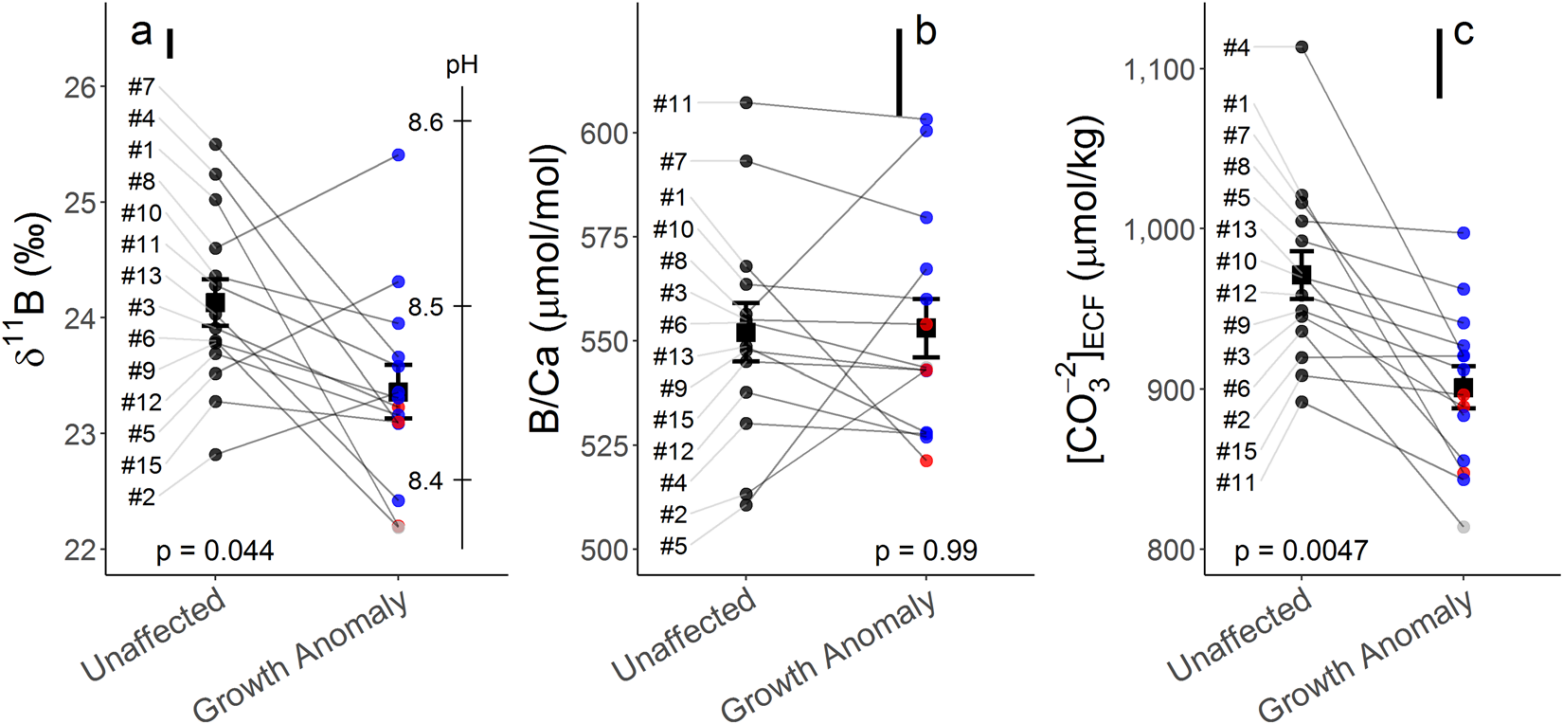
Skeletal measurements of boron systematics and associated estimates of internal carbonate chemistry from unaffected and growth anomaly *Porites compressa*. Average and individual measurements of δ^11^B along with pH_ECF_ (**a**), B/Ca ratio (**b**) and [CO_3_^2−^]_ECF_ (**c**) are shown for unaffected (n = 14) and growth anomaly (n = 14) samples. Black squares represent group mean (± standard error of the mean) and black (unaffected), blue (Form 1), red (Form 2) and grey (unclassified) circles indicate individual sample morphology. Sample numbers are indicated along the left margin and grey lines connect each paired set of unaffected and growth anomaly samples. Floating black bars indicate analytical uncertainty for δ^11^B, B/Ca and [CO_3_^2−^]_ECF_. Presented p-values result from paired sample t-tests.

In addition to consistent observable macro-morphological differences, we also detect differences in multiple chemical measurements between Form 1 and Form 2 lesions (Supplementary Table S1). This includes trends of lower B/Ca, δ^11^B, pH_ECF_, [CO_3_^2^]_ECF_, and higher U/Ca in Form 2 compared to Form 1 GAs, where both forms tend to differ from unaffected skeleton chemical properties in the same direction, with a larger magnitude of difference for Form 2 GAs (Fig. 3, Fig. 4). These metrics are closely tied to coral calcification (mechanisms discussed below), lending validity to the separation of these two growth forms. Subdividing our samples led to uneven replication of Form 1 (n = 10) and Form 2 (n = 3) lesions, hence, to confirm our findings, we recommend further sampling expeditions and characterization of these forms at the cellular/molecular level. Identification of Form 2 as a truly separate GA type will further our understanding of disease dynamics in *P. compressa*.

### Centers of calcification

Average geochemical metrics for paired GA and unaffected skeletons are summarized in Table 2, and are similar to Mg/Ca (4.9–5.2 mmol/mol) but higher than Sr/Ca (7.6–7.9 mmol/mol) previously measured for *P. compressa*^17^. We report higher U/Ca (1.219 ± 0.044 vs 1.060 ± 0.032 µmol/mol, p = 0.0014) and lower Mg/Ca (4.70 ± 0.07 vs 5.14 ± 0.08 mmol/mol, p = 0.0005) in GA (n = 14) compared to paired unaffected (n = 14) samples (Fig. 3). Rayleigh fractionation, growth entrapment and kinetic models would predict enhanced inclusion of otherwise incompatible elements into the skeleton in the faster growing GA specimens, resulting in higher Mg/Ca and lower U/Ca in GAs^26,34,35^. Here, we observe the opposite trends, suggesting that growth rate effects do not explain the Mg/Ca and U/Ca results. Therefore, alternative explanations for these elemental differences are considered below.

Coral skeletons are composed of micro-structural elements that dictate coral macro-morphology including centers of calcification (COC) where nucleation begins, and fibrous crystals which subsequently radiate out from these centers. Observations in healthy corals show COC are enriched in Mg while having lower U compared to the extending fibrous aragonite crystals^29,36,37^. Lower Mg/Ca and higher U/Ca ratios in GAs could therefore be a result of a decrease in the relative abundance of COC or alterations in COC chemistry (decreased Mg/Ca and increased U/Ca). Because of their importance in the initial calcification process, irregular COC abundance or composition in GAs could contribute to aberrant skeleton growth.

Scanning electron microscopy (SEM) images of polished coral sections show skeletal microarchitecture (Fig. 1b, Supplementary Fig. S3), however, as has been previously reported in other *Porites* spp.^38^, COC are not clearly visible. Therefore, we are unable to quantify COC abundance using SEM or light microscopy, as one could with cold water corals with larger COC^29^. This precludes us from testing the hypothesis that decreased abundance of Mg-rich COC contributes to low Mg/Ca seen in GAs. Despite identical treatment of the samples, polished surfaces of GA skeletons look completely different. Contrast the robust and thick trabeculae of unaffected skeleton that have a homogenous texture on cut to that of GA with more abundant thinner, chaotic to meandering to serpiginous trabeculae with a cut surface composed of haphazardly arranged stellate crystals suggestive of aragonite fibers. This supports our hypothesis that skeleton composed of relatively fewer COC and more fibrous crystals is the best explanation for low Mg/Ca we observe in GAs.

### Carbonate chemistry in the extracellular calcifying fluid

Deep-sea corals have lower U/Ca and δ^11^B in COC relative to surrounding growing aragonite fibers^39^. However, unlike U/Ca, we report lower δ^11^B in *P. compressa* GAs, which would not be explained by the theorized decreased abundance of COC. Carbonate system controls on boron systematics are therefore discussed below.

δ^11^B and B/Ca data indicate that pH_ECF_ and [CO_3_^2−^]_ECF_ in GAs (n = 14) are respectively 0.05 units (p = 0.044) and 70 µmol/kg (p = 0.0047) lower on average relative to paired unaffected (n = 14) samples (Fig. 4). The δ^11^B can be highly variable within a single coral^39^, which may help explain why some pairs show smaller pH decreases from unaffected to GA samples. Nonetheless, many pairs (e.g. #1, 4, 6, 7, 13; Supplementary Table S2) have notably large internal pH differences of over 0.1 units between GA and unaffected specimens. The increased U/Ca (proxy with an inverse relationship with [CO_3_^2−^]_ECF_^37,40^) in GAs corroborates our finding of decreased [CO_3_^2−^]_ECF_ derived from boron systematics. If COC are lower abundance in GAs as hypothesized, internal pH in GA skeleton is likely overestimated here due to the relative increase in high-δ^11^B fibers, meaning pH upregulation may be compromised to an even greater extent.

Decreased pH_ECF_ and [CO_3_^2−^]_ECF_ may be explained by rapid growth in GAs compared to unaffected samples. Accelerated calcification could theoretically decrease pH_ECF_ and [CO_3_^2−^]_ECF_, if ion pumping fails to keep pace replacing precipitated ions in the calcifying fluid. However, healthy coral studies show that calcification rates do not correlate with internal aragonite saturation state^41^, and skeleton precipitation mass does not correlate with U/Ca^42^. These studies suggest that calcification rates have little impact on internal carbonate parameters. By contrast, lower internal saturation states have been linked to decreased skeletal density^41^. Decreased density in *P. compressa* GAs has been previously documented^17^, and our SEM images show multiple pits and holes in the GA skeleton (Fig. 1b, Supplementary Fig. S3). These defects possibly arise as artifacts during initial calcification or from skeleton-boring microorganisms such as cyanobacteria, fungi, green and red algae and provide further evidence of a fragile and porous GA crystal structure. Furthermore, previous studies have concluded that, despite higher skeletal extension rates, calcification rates are not elevated in *P. compressa* GAs^17^. We therefore suggest that decreased pH_ECF_ and [CO_3_^2−^]_ECF_ measured here are unrelated to calcification rates.

Alternatively, lower internal pH and [CO_3_^2−^] may be a result of decreased physiological upregulation of these parameters in GAs. *P. compressa* GAs have fewer symbiotic dinoflagellates compared to surrounding tissues^17^, presumably limiting their available energy budget. GAs therefore impose energetic demands on the surrounding tissues to help sustain their elevated growth^16^. Furthermore, Domart-Coulon *et al*.^17^ theorized that impaired gonad development they observed in GAs was also a result of localized energy allocation towards tissue growth at the expense of reproduction. Maintaining an upregulated pH_ECF_ is an energy-consuming process^25^, therefore it is possible that the decreased pH_ECF_ we measure in GAs is due to energy re-allocation away from pH upregulation and towards tissue growth. In fact, in some cases coral bleaching (expulsion of symbiotic dinoflagellates) corresponds with similar decreases in δ^11^B^43–45^, further demonstrating a link between energy availability and ECF regulation. Such a case could negatively impact the quality of the growing skeleton as aragonite is precipitated from lower saturation state conditions, contributing to the less dense, irregular skeleton characteristic of GAs.

Although the underlying mechanisms require further elucidation, the fundamental differences we observe in the ECF chemistry provide new insights into the pathophysiology of the disease and uncovers a new mechanism of interest (poor physiological pH_ECF_ upregulation) that may be linked to the irregular skeletal growth in GAs. The ability to upregulate pH_ECF_ is critical for coral resilience to changes in ambient seawater pH^28^, therefore, lower internal pH and porous skeleton structure in GAs indicate that these lesions will be particularly susceptible to the increasing global threat posed by ocean acidification. Under such conditions, GAs must either increase energy allocated towards pH_ECF_ upregulation, precipitate their skeleton from increasingly acidic internal conditions, or decrease calcification rates leading to increased skeleton porosity. Although these impacts alone may not be likely to threaten coral populations, as GA prevalence for *Porites* species across the Indo-Pacific averages just 0.2%, topping out at 16.7% in the Hawaiian Islands^23^, if GA prevalence increases in future populations they may compound with previously documented deleterious effects of GAs to considerably increase the ecological threat that they pose.

### Essential trace elements

Carbonic anhydrase (CA) enzymes facilitate the interconversion of carbon dioxide and bicarbonate and play an important role in coral calcification^46^. Typically, Zn^2+^ ions act as the metal co-factor to CA, but Co^2+^ can substitute as a co-factor resulting in native enzymatic properties^47^. Substitutions may also occur with Cu^2+^ and Mn^2+^ ions acting as co-factors, however the resulting enzyme no longer retains its native conformation^47^. The lack of difference between GA and unaffected samples for all of these trace metals (Table 2, p > 0.05) provides no support for disruption of carbonic anhydrase activity in GAs.

**Figure 5:**
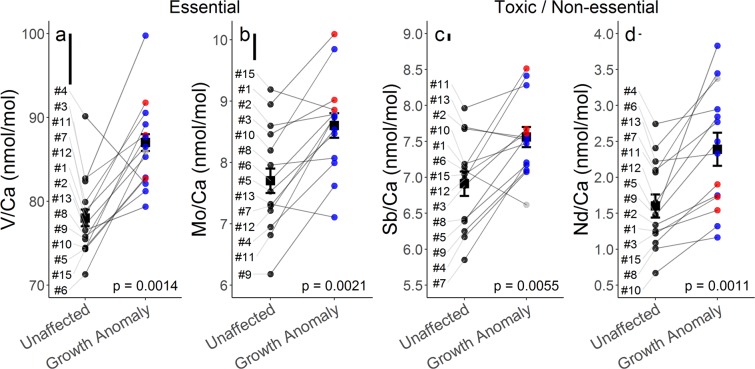
Skeletal measurements of selected essential and toxic/non-essential trace metals from unaffected and growth anomaly *Porites compressa*. Average and individual measurements of essential V/Ca (**a**) and Mo/Ca (**b**) as well as non-essential Sb/Ca (**c**) and Nd/Ca (**d**) ratios are shown for unaffected (n = 14) and growth anomaly (n = 14) samples. Black squares represent group mean (±standard error of the mean) and black (unaffected), blue (Form 1), red (Form 2) and grey (unclassified) circles indicate individual sample morphology. Sample numbers are indicated along the left margin and grey lines connect each paired set of unaffected and growth anomaly samples. Floating black bars indicate analytical uncertainty for V/Ca, Mo/Ca, Sb/Ca and Nd/Ca. Presented p-values result from paired sample t-tests for V/Ca, Mo/Ca and Sb/Ca and from a Wilcoxon signed-rank test for Nd/Ca.

We do however report higher Mo/Ca (8.6 ± 0.2 vs 7.7 ± 0.2 nmol/mol, p = 0.0021) and V/Ca (87 ± 1 vs 78 ± 1 nmol/mol, p = 0.0014) in GA (n = 14) compared to unaffected (n = 14) samples (Fig. 5). Molybdenum and Vanadium are co-factors in nitrogenase enzymes commonly belonging to nitrogen-fixing bacteria and archaea, and these enzymes may account for a majority of diazotrophic biological requirements for Mo^48,49^. Higher efficiency Mo-nitrogenases are generally preferred by organisms over V-nitrogenases, however both forms can contribute towards total nitrogen-fixation^50^. Vanadium is also linked to nitrogen fixation through its role as a co-factor in haloperoxidase enzymes which neutralize reactive oxygen species that acutely inhibit nitrogenase activity^48,51^. Corals host diverse communities of microbes throughout the coral holobiont both between species and within a coral individual^52–54^. These diazotrophs are found throughout coral mucus, tissues, and skeleton and contribute nitrogen towards coral holobiont nutrition^52,55,56^. The most conspicuous holobiont alteration associated with GAs is the reduction in symbiotic dinoflagellates^15^ but altered microbial community composition and function are also associated with *P. compressa* GAs^57^.

We therefore theorize that Mo and V are accumulating in GA skeletons as a function of nitrogen-fixation in GA-associated diazotrophs. Indeed, recent research suggests that skeletal Mo may act as a proxy for coral holobiont biological activity^58^. A relative decrease in biological sequestration of these metals from the ECF of GAs for nitrogen metabolism, such as by diazotrophs residing in the coral tissues, may therefore result in the higher skeletal Mo/Ca and V/Ca we observe. If correct, a decrease in nitrogen-fixation activity provides further evidence for compromised energetics in these diseased corals, which is likely linked to their lower abundance of symbiotic dinoflagellates. The decreased abundance of endosymbiotic dinoflagellates may also contribute towards higher Mo/Ca in GAs through a similar mechanism, as less Mo is collectively taken up for metabolic processes such as nitrogen assimilation^58^. Our results highlight the possibility of Mo/Ca and V/Ca as proxies for bacterial nitrogen-fixation, however we encourage future interdisciplinary work (e.g.^59^.) to better characterize holobiont functioning that includes microbial DNA sequencing of GA specimens to confirm this link and hypothesized metal incorporation pathway.

### Toxic and non-essential elements

We also detect higher Nd/Ca (2.39 ± 0.23 vs 1.60 ± 0.16 nmol/mol, p = 0.0011) and Sb/Ca (7.56 ± 0.14 vs 6.91 ± 0.17 nmol/mol, p = 0.0055) in GA (n = 14) compared to unaffected (n = 14) samples (Fig. 5). Our average elemental values for *P. compressa* fall within the range of Nd/Ca (1.4 to 3.3 nmol/mol^60^) but are lower than Sb/Ca (30.8 to 113 nmol/mol^61^), reported for other shallow-water corals. Although the uptake mechanism of these elements remains poorly understood, elevated Nd/Ca and Sb/Ca in GAs (Fig. 5) (despite exposure to identical seawater [Nd] and [Sb]) perhaps indicates these lesions have reduced control over the uptake from seawater and/or the incorporation into the skeleton of these elements. Acute toxicity to aquatic organisms of both Sb and Nd vary by orders of magnitude by species, and toxicity data for Nd are particularly sparse^62,63^, making toxicological interpretations of the increased Nd/Ca and Sb/Ca we see in *P. compressa* GAs difficult. Nonetheless, the environmental effects of Sb and Nd from industrial sources are of growing concern^62,63^. Therefore, considering the linkage between GA prevalence and human population density, a role for these elements in GA formation should not be discounted, and future studies of the toxicity of these elements to corals may provide valuable insights to this end.

## Conclusion

In this study, we provide a novel combination of detailed morphological and skeleton elemental descriptions of GAs in reef-building *P. compressa* coral specimens. SEM images and chemical results further demonstrate the fragile nature of GA skeleton, which in addition to high porosity and incidence of skeletal defects, may have fundamental differences in the abundance of critical skeletal microstructural elements (COC) related to skeletal pathophysiology. Boron systematics also reveal that GAs have decreased internal pH and [CO_3_^2−^], parameters intimately linked to coral calcification. We theorize growth demands in GAs may help explain GA carbonate system parameters, as energy is allocated towards tissue growth at the expense of pH_ECF_ upregulation. Collectively, our results highlight the compromised calcification and skeleton structure of GAs, and the energetic burden their growth places on affected specimens thus lowering their overall fitness.

## Methods

### Sample collection

Coral samples were collected within a 1 hr period in March 2014 from living *P. compressa* colonies from depths less than 3 m (State of Hawaii Division of Aquatic Resources Special Activity Permit 2011-1). Samples (approximately 4 cm in diameter) of two treatment groups were collected from individual diseased colonies (n = 14): coral fragments exhibiting GAs (GA, n = 14) and apparently healthy fragments directly adjacent to the anomalous growth on the same colony (unaffected, n = 14), resulting in a set of paired GA and unaffected samples. Once brought to the surface, samples were frozen immediately in liquid nitrogen to preserve the physiological state of the samples.

### Morphological characterization

Samples were transferred from liquid nitrogen and freeze-dried in a VirTis Genesis OX lyophilizer with a Wizard 2.0 controller (SP Industries Inc., Warminster USA). For GAs, lesion macro-morphology descriptions were made of the edges, shape, and relief for each GA lesion generally following terminology by Work and Aeby^12^. Physical measurements of GA lesion size (length × width × height) and depth of tissue layer into the coral skeleton (average of 5 measurements) were made for each sample using Vernier calipers. For observations of skeletal morphology, samples were imaged using an Olympus DP-71 digital microscope camera equipped with cellSens imaging software (Olympus Corp., Tokyo Japan) to calculate corallite diameter (average of 5 measurements) and corallite spatial density (average of three 5 × 5 mm digital quadrats) for each sample. Finally, skeleton micro-architecture was assessed using representative skeletal nubbins transversely sectioned using a diamond saw blade and polished with fixed abrasive silicon carbide paper. Prepared nubbins were coated in 100 Å of gold-palladium and imaged by scanning electron microscopy.

### Sample processing

GA lesions were separated by hammer and stainless-steel chisel from surrounding unaffected tissue before a scalpel was used to collect skeletal powders for elemental analysis by scraping to the full tissue depth into the skeleton from the apical area of the lesion or unaffected fragment. The skeleton/tissue powder was then subject to established cleaning procedures to remove organic matter^64,65^. Powders were agitated in 6% laboratory grade sodium hypochlorite solution (Thermo Fisher Scientific Inc., Waltham USA) for at least 12 hr before rinsing with high-purity deionized water (Boron Guard Cartridge; >18.2 MΩ-cm). Subsamples (~5 mg) were further oxidatively cleaned using buffered 1% H_2_O_2_ and then given a weak acid leach (0.0005 M HNO_3_) to remove any re-adsorbed ions.

Lesions in *P. compressa* are often transient (i.e. present for ~1 year)^16^ hence we selected recently deposited skeleton for geochemical analysis to ensure samples definitively represent GA calcification. However, because skeletal powders were collected from the layer occupied by coral organic tissues, geochemical results have potential to be influenced by residual organics which may bias results. This is of particular concern given the differential tissue characteristics in unaffected and GA samples which potentially react differently to cleaning protocols. Therefore, we implement a thorough, two-step cleaning protocol (compared to single-step protocols such as in^66^) that is demonstrated to effectively remove organics^67^. Furthermore, we show that Li/Mg ratios (a temperature proxy prone to organic matter contamination) in our unaffected corals fall within the Li/Mg range of published tropical corals living at similar sea surface temperature (Supplemental Fig. S4). Additionally, GA-unaffected trends for organic-sensitive elements (e.g. Mg and U) are similar regardless of sampling in organic-rich or -poor regions of the coral (Supplemental Fig. S5). Together, these results suggest that our cleaning protocols are effective, and samples are minimally impacted by residual tissue.

After cleaning, samples were then dissolved in a minimum volume of Optima^TM^ 0.5 M HNO_3_ (Thermo Fisher Scientific Inc., Waltham USA). All chemical analyses were carried out at the National Institute of Standards and Technology (Charleston, SC).

### Trace element analysis

Trace element ratios (to calcium) were measured on a small aliquot (<10%) of the sample solutions on a Thermo Element II ICP-MS (Thermo Fisher Scientific Inc., Waltham USA). Sample solutions were screened for calcium concentration and diluted in 0.5 M HNO_3_ to 80 µg/g [Ca] for analysis using a method modified from Marchitto^68^. Multi-element external calibration using gravimetrically prepared matrix-matched standards were used to quantify Li, B, Na, Mg, Al, V, Mn, Fe, Co, Ni, Cu, Zn, Rb, Sr, Mo, Sb, Ba, Nd, Pb and U analytes. Elemental counts were blank corrected using a blank acid run after each sample/standard. The percent relative standard deviation (%RSD; 1σ) was calculated for each elemental ratio from replicate measurements (n = 37) of a matrix-matched control material (NIST RM 8301-Coral) to assess analytical precision. The majority of the measured trace elements had uncertainty ≤ ±2% (Li/Ca, Na/Ca, Mg/Ca, Al/Ca, Co/Ca, Cu/Ca, Rb/Ca, Sr/Ca, Sb/Ca, Ba/Ca, Nd/Ca, Pb/Ca and U/Ca), with B/Ca, Fe/Ca, Ni/Ca, Zn/Ca ≤ ±5%, and V/Ca, Mn/Ca, Mo/Ca ≤ ±10%.

### Boron isotope analysis

Boron isotope analysis broadly followed the protocols of Foster^69^ and Rae *et al*.^70^. Boron in the remaining dissolved skeletal solutions (≈200 ng of B) was separated from the carbonate matrix using 20 µL micro-columns containing Amberlite IRA 743 boron-specific anionic exchange resin^71^. Following elution of the boron fraction, additional elutions were checked to ensure >99% of sample boron was recovered in the sample. The purified boron samples were diluted to 100 ppb [B] for analysis and were measured in duplicate on a Nu Plasma II MC-ICP-MS (Nu Instruments Ltd., Wrexham UK) against matrix-matched solution of NIST SRM 951a. An on peak zero was acquired as a 60 s acid blank measurement before each sample/standard. Seven total procedural blank measurements made alongside samples were found to be small (average of 104 pg B; i.e. <0.06% of sample) resulting in minimal impact on δ^11^B sample results (i.e. less than analytical uncertainty), hence a total procedural blank correction was not applied. Carbonate standards JCp-1 (24.2‰^72^) and NIST RM 8301-Coral (24.2‰^73^) measured during sample analysis gave values of 24.06‰ (n = 2), and 24.35‰ (n = 34) ±0.26‰ respectively.

### ECF carbonate system parameter calculations

The pH_ECF_ was estimated from measured skeletal δ^11^B according to Eq. (1) from Dickson^74^:

1 $${{\rm{pH}}}_{{\rm{ECF}}}={\rm{p}}{K}_{B}^{\ast }-\,\log \left(-\frac{{{\rm{\delta }}}^{11}{{\rm{B}}}_{{\rm{sw}}}-{{\rm{\delta }}}^{11}{{\rm{B}}}_{{\rm{coral}}}}{{{\rm{\delta }}}^{11}{{\rm{B}}}_{{\rm{sw}}}-{{\rm{\alpha }}}_{B}{{\rm{\delta }}}^{11}{{\rm{B}}}_{{\rm{coral}}}-{1000({\rm{\alpha }}}_{B}-1)}\right)$$

where α_B_ (1.027) is the fractionation factor between boric acid and borate^75^, δ^11^B_sw_ (39.61) is the δ^11^B of seawater^76^ and pK_B_^*^ (8.6) is the dissociation constant of the two boron species calculated using site-specific temperature (25.0 °C) and salinity (35.0 psu), based on average measurements at the CRIMP2 monitoring station between years 2009 and 2015^77^). Analytical uncertainty on δ^11^B measurements contributes to a <0.02 pH unit shift in estimated internal pH. Skeletal B/Ca varies as a function of [CO_3_^2−^] in the ECF according to Eq. (2):

2 $${[{{\rm{CO}}}_{3}^{2-}]}_{{\rm{ECF}}}={D}_{{\rm{B}}/{\rm{Ca}}}\frac{{[{\rm{B}}({\rm{OH}}{)}_{4}^{\mbox{--}}]}_{{\rm{ECF}}}}{{{\rm{B}}/{\rm{Ca}}}_{{\rm{Aragonite}}}}$$

where *D*_B/Ca_ is the partition coefficient of boron into aragonite from seawater (i.e. [B/Ca]_Aragonite_/[B/Ca]_Seawater_). [B(OH)_4_^−^]_ECF_ is calculated using Eq. (3):

3 $$[{\rm{B}}({\rm{OH}}{)}_{4}^{\mbox{--}}]=\frac{{{\rm{B}}}_{T}}{{1+[{\rm{H}}}^{+}]{\rm{ECF}}/{K}_{B}^{\ast }}$$

where B_T_ is the total boron in seawater (432.5 µmol/kg at salinity 35 psu^78^) and [H^+^]_ECF_ is estimated using skeletal δ^11^B-pH proxy data and Eq. (1). In this way, a second carbonate system parameter [CO_3_^2−^]_ECF_ can be calculated from paired skeletal δ^11^B and B/Ca data using the method of DeCarlo *et al*.^32^. Here we use D_B/Ca_ formulations and dissociation constants of carbonic acid by McCulloch *et al*^31^. and Lueker *et al*^79^. respectively. Monte Carlo simulations (1000 iterations) factoring in analytical errors on δ^11^B and B/Ca suggest uncertainty on these [CO_3_^2−^]_ECF_ estimates of <±53 µmol/kg.

### Statistical analysis

Paired sample t-tests (2-tailed) were used to test for differences in morphological and chemical metrics between paired GA and unaffected samples. Where the differences between paired samples were not normally distributed (Na/Ca, Ba/Ca), an alternative non-parametric Wilcoxon signed-ranked test (2-tailed) was used. This test was further used for any elements with samples below the limit of detection (Fe/Ca, Co/Ca, Nd/Ca), with all non-detects assigned as ties at the limit of detection (average of the blanks plus 3σ). For these elements, a robust regression on order statistics was used to estimate group mean and standard error on the mean using the R *NADA* package (R 3.6.1). All p-values were adjusted using Benjamini-Hochberg false discovery rate^80^ to account for the multiple comparisons.

## Supplementary information

Supplementary Materials.

Supplementary Table S2.

## Data Availability

All data for individual coral morphological and geochemical measurements are available in the Supplementary information.
