# Supplementary material for: Morphological, elemental, and boron isotopic insights into pathophysiology of diseased coral growth anomalies
Source: Sci Rep. 2020 May 19;10:8252. doi: 10.1038/s41598-020-65118-6 (PMC7237652; doi:10.1038/s41598-020-65118-6)
Supplement: Supplementary file 1 — Supplementary Materials. [file 41598_2020_65118_MOESM1_ESM.pdf]

Supplementary Materials for

Morphological, elemental, and boron isotopic insights into pathophysiology of diseased coral growth anomalies

Erik R. Andersson[1,2*], Joseph A. Stewart[2,3], Thierry M. Work[4], Cheryl M. Woodley[5], Tracey B. Schock[2], and Rusty D. Day[2,6]

[1] Grice Marine Laboratory, Department of Biology, College of Charleston, 205 Fort Johnson Rd., Charleston, SC 29412, USA

[2] Marine Biochemical Sciences, Chemical Sciences Division, National Institute of Standards and Technology, Hollings Marine Laboratory, Charleston, SC 29412, USA

[3] School of Earth Sciences, University of Bristol, Queens Road, Bristol, BS8 1RJ, UK

[4] U.S. Geological Survey National Wildlife Health Center, Honolulu Field Station, Honolulu, HI 96850, USA

[5] Hollings Marine Laboratory, National Ocean Service, National Oceanic and Atmospheric Administration, Charleston, SC 29412, USA

[6] Current address: Marine Science and Nautical Training Academy (MANTA), 520 Folly Rd., Charleston, SC 29412, USA

*Correspondence: anderssoner@g.cofc.edu

Supplementary Figure S1. Map of Kaneohe Bay, Oahu, Hawaii. Red box indicates sample collection location. Colors indicate percentage coral cover of all coral species [1,2]. Map was generated using Ocean Data View [3] with Google Earth Satellite overlay [4].

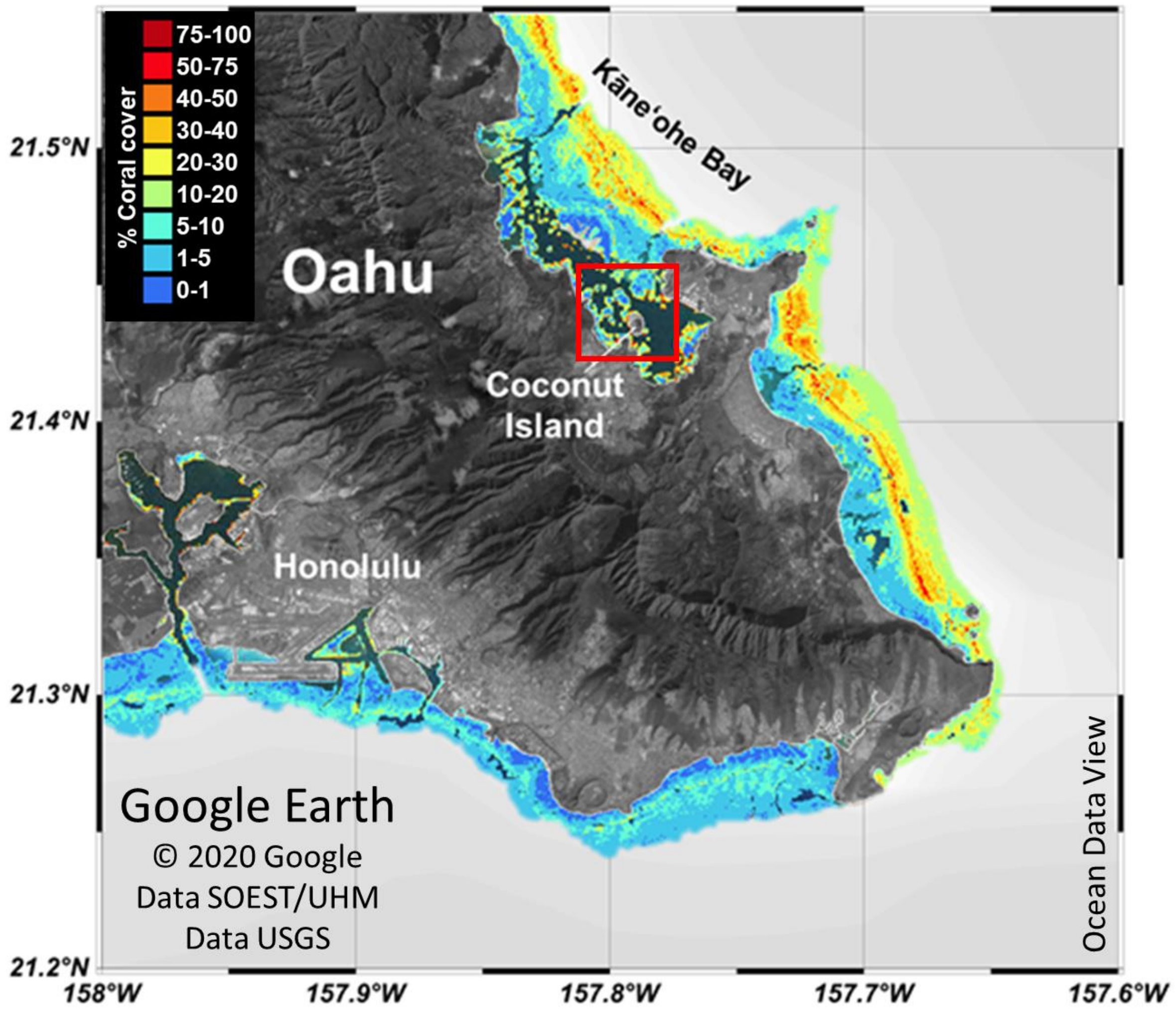

Supplementary Figure S2. Growth anomaly (GA) lesion macro-morphologies of samples not shown in the primary text. All samples shown here were classified as Form 1 lesions, except for sample 6 GA (could not be determined). Dashed red lines indicate GA lesion area.

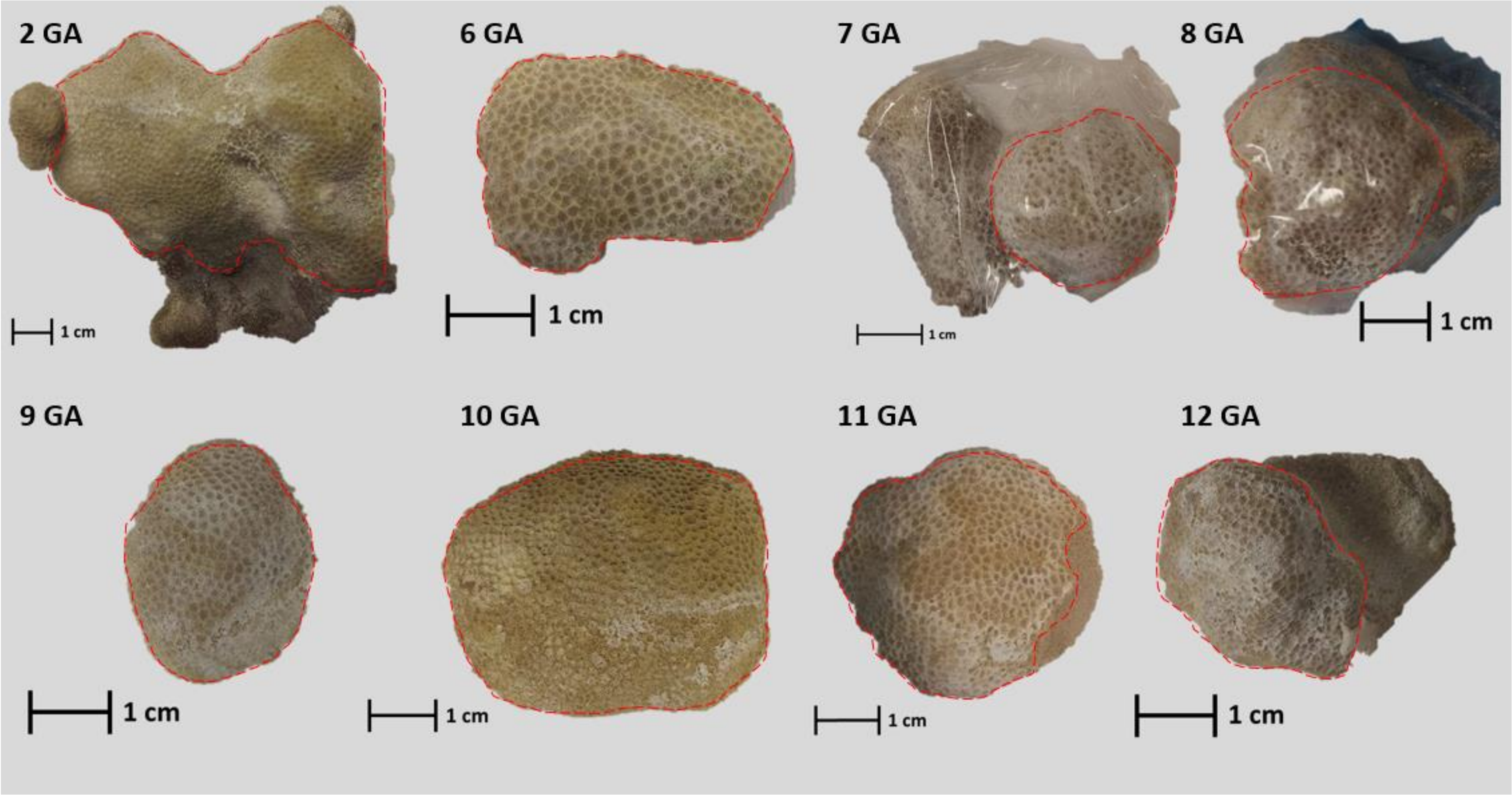

Supplementary Figure S3. Representative scanning electron microscopy images of growth anomaly (GA) and unaffected (U) *Porites compressa* skeleton.

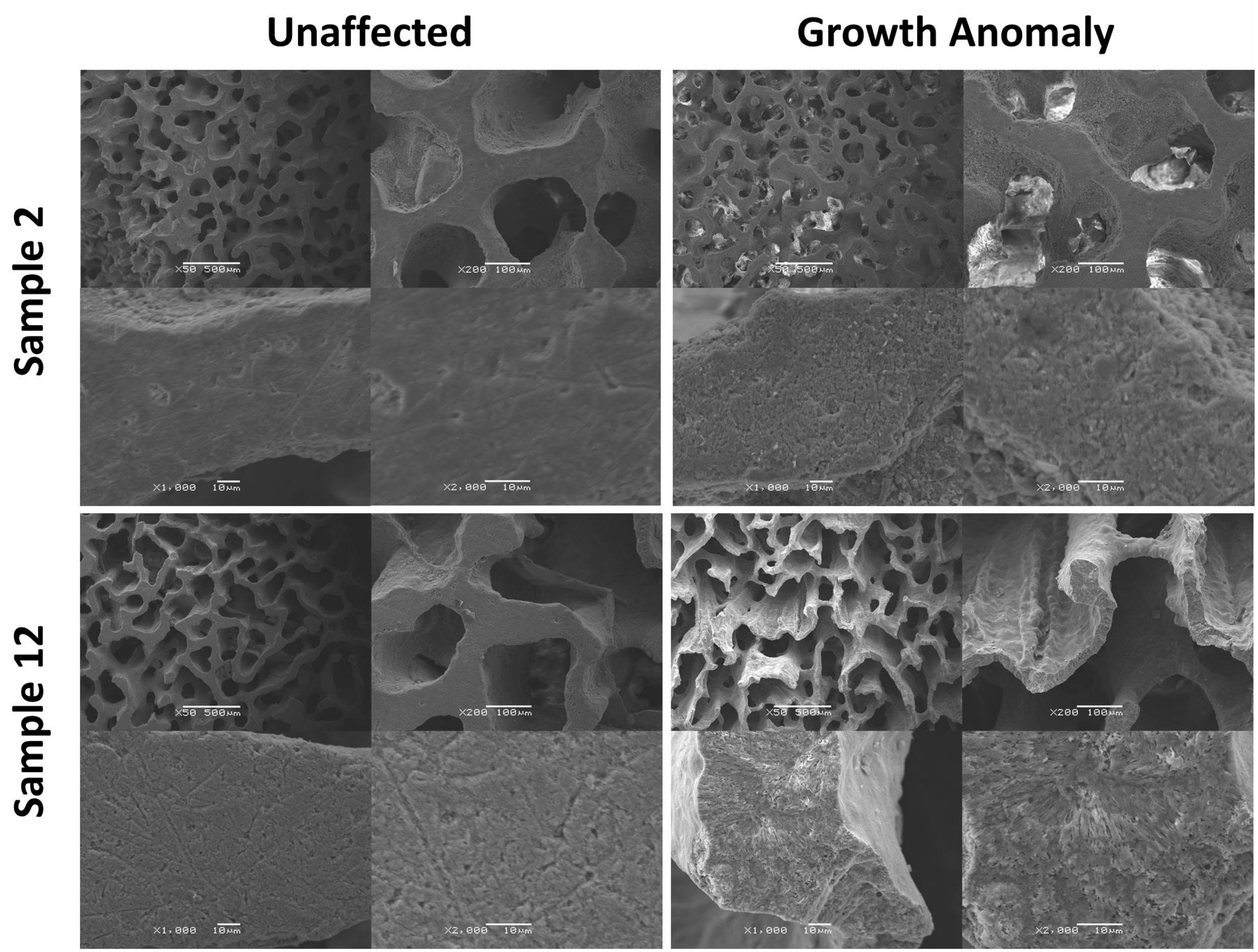

Supplementary Figure S4. Li/Mg values as a function of seawater temperature from unaffected samples in this study (red diamonds) and tropical corals from the literature (black circles). Published data are from [5-9] and have been complied and adjusted for interlaboratory analytical offsets by [10]. For visualization and comparability of results in this figure, all Li/Mg values from this study were multiplied by a factor of 1.1 to bring Li/Mg values of the coral reference material JCp-1 (1.33 mmol/mol; this study) into line with inter-laboratory results (1.47 mmol/mol; [11]).

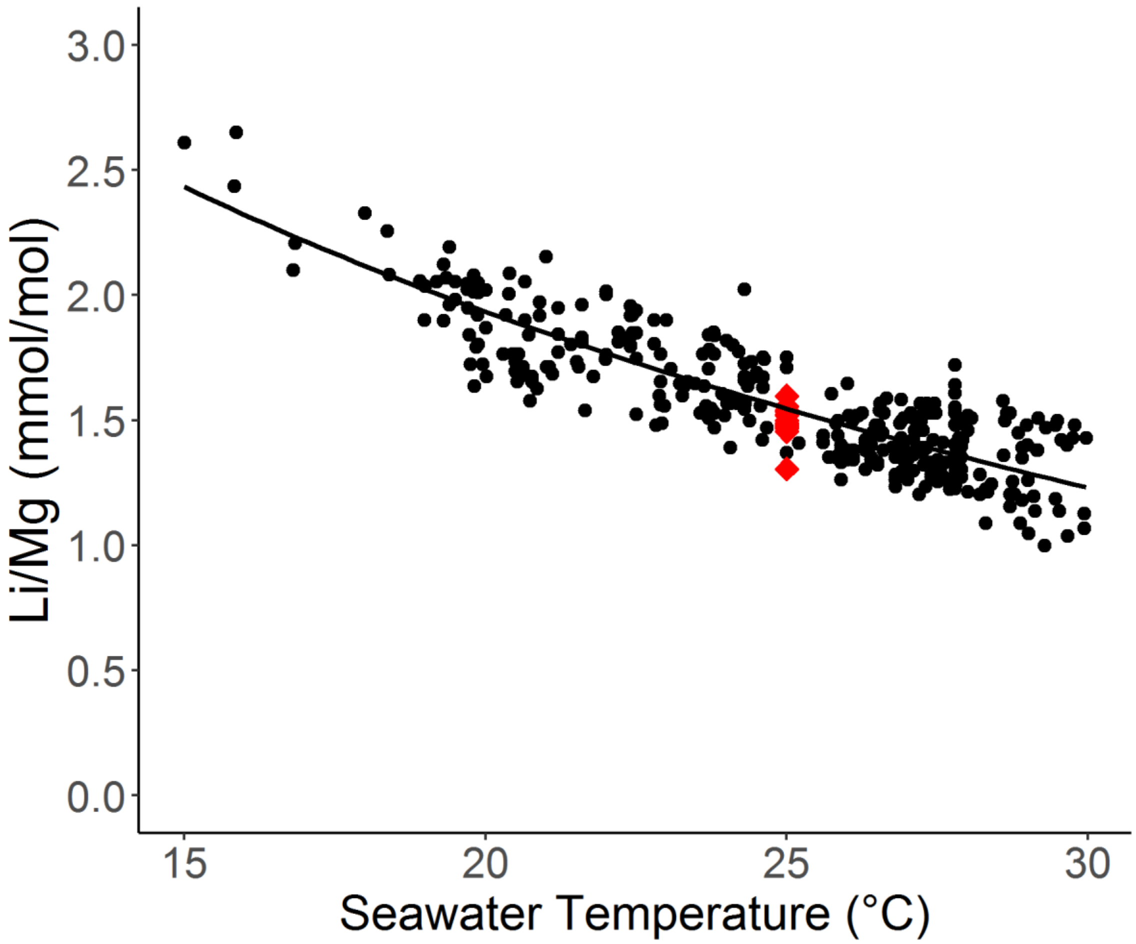

Supplementary Figure S5. Element to calcium ratios for elements of concern for potential residual organic contamination. Results are triplicate measurements from paired unaffected and growth anomaly *Porites compressa* samples. Triplicate measurements (black points) are shown from: an unaffected sample from the organic-rich mixed tissue/skeleton layer that was used in this study (U Mixed); from the organic-poor deeper skeleton layer of the same sample (U Skeleton); a GA sample from the organic-rich mixed tissue/skeleton layer (GA Mixed); and from the organic-poor deeper skeleton layer of the same sample (GA Skeleton). Dashed lines separate the mixed layer values from the skeleton layer values for each trace metal. * indicates second Sb/Ca plot where one outlier GA Skeleton sample (135.26 nmol/mol) was removed for visualization purposes.

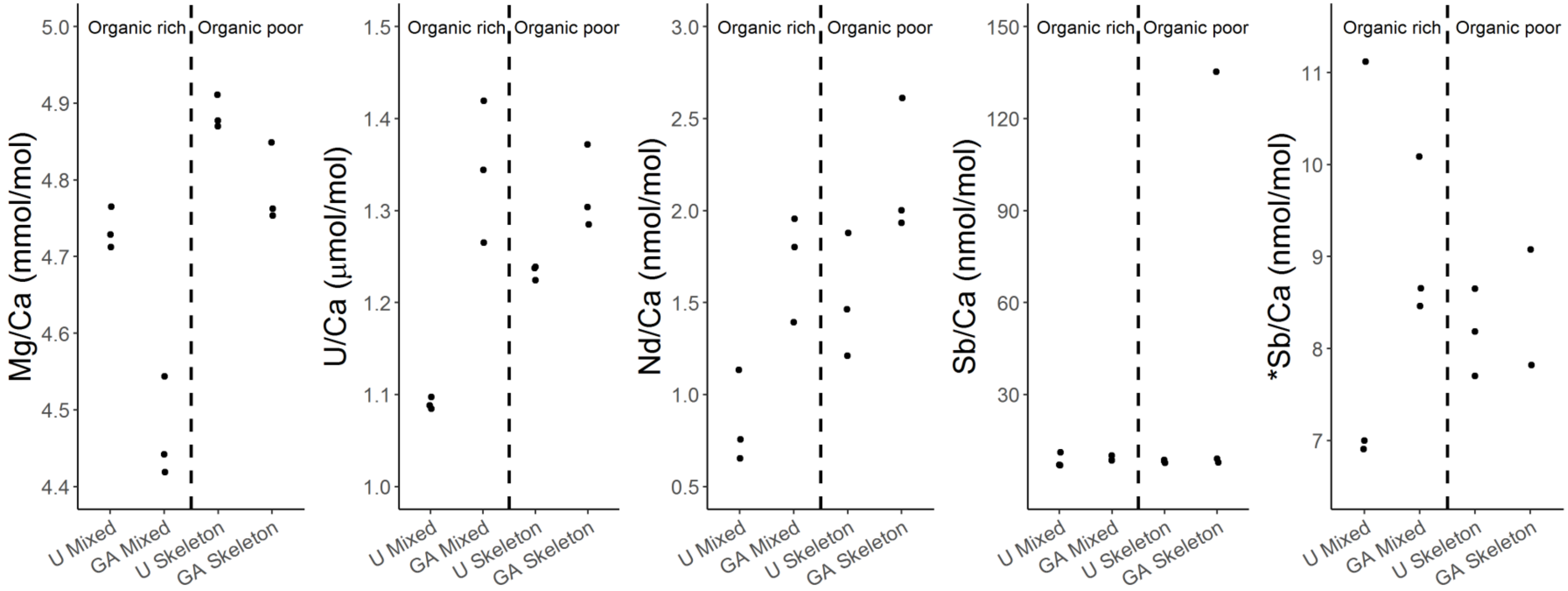

Supplementary Table S1. Mean (±SE) morphological and chemical measurements for Form 1 (n=10) and Form 2 (n=3) growth anomaly lesions. For elements with non-detects, a robust regression on order statistics was used to estimate group mean and standard error. Internal pH is calculated from $\delta^{11}B$ using formula by [12] and $[CO_3^{2-}]_{ECF}$ is calculated from $\delta^{11}B$ and B/Ca using method of [13].

		Form 1	(± SE)	Form 2	(± SE)
**Morphology**					
Tissue Depth	mm	4.5	0.2	4.6	1.3
Corallite Spatial Density	$\#/mm^2$	0.66	0.03	0.62	0.15
Corallite Diameter	mm	1.47	0.05	1.57	0.19
**Trace Elements**					
Li/Ca	(µmol/mol)	6.77	0.08	6.89	0.09
B/Ca	(µmol/mol)	558	9	539	10
Na/Ca	(mmol/mol)	21.92	0.23	23.20	0.07
Mg/Ca	(mmol/mol)	4.72	0.09	4.68	0.15
Al/Ca	(µmol/mol)	8.24	1.29	7.00	1.30
V/Ca	(nmol/mol)	86	2	87	3
Mn/Ca	(µmol/mol)	0.91	0.08	1.06	0.03
Fe/Ca	(µmol/mol)	1.73	0.28	1.72	0.10
Co/Ca	(µmol/mol)	0.468	0.007	0.464	0.002
Ni/Ca	(µmol/mol)	5.54	0.03	5.46	0.03
Cu/Ca	(µmol/mol)	0.305	0.024	0.130	0.035
Zn/Ca	(µmol/mol)	29.6	5.4	12.3	4.4
Rb/Ca	(nmol/mol)	10.78	0.19	10.75	0.32
Sr/Ca	(mmol/mol)	9.40	0.05	9.43	0.05
Mo/Ca	(nmol/mol)	8.4	0.2	9.3	0.4
Sb/Ca	(nmol/mol)	7.54	0.15	7.93	0.29
Ba/Ca	(µmol/mol)	41.40	9.09	14.29	9.03
Nd/Ca	(nmol/mol)	2.49	0.28	1.72	0.10
Pb/Ca	(nmol/mol)	14.09	1.87	4.45	0.24
U/Ca	(µmol/mol)	1.178	0.045	1.380	0.113
**Boron Systematics**					
$\delta^{11}B$	(‰)	23.63	0.26	22.84	0.32
pH_{ECF}		8.47	0.02	8.42	0.02
$[CO_3^{2-}]_{ECF}$	(µmol/kg)	916	15	878	15

References

1. Franklin, E. C., Jokiel, P. L. & Donahue, M. J. Predictive modeling of coral distribution and abundance in the Hawaiian Islands. *Mar. Ecol. Prog. Ser*. **481**, 121–132, doi:10.3354/meps10252 (2013).
2. Franklin, E. C., Jokiel, P. L. & Donahue, M. J. Data from: Predictive modeling of coral distribution and abundance in the Hawaiian Islands. *Dryad Digital Repository*, doi:10.5061/dryad.9vd0q (2013).
3. Schlitzer, R. *Ocean Data View*, URL:https://odv.awi.de, v5.2.1 (2020).
4. Map of Kaneohe Bay, Oahu, Hawaii, United States. *Google Earth*, URL:earth.google.com/web/, v9.3.106.3 (2020).
5. Hathorne, E. C., Felis, T., Suzuki, A., Kawahata, H. & Cabioch, G. Lithium in the aragonite skeletons of massive *Porites* corals: A new tool to reconstruct tropical sea surface temperatures. *Paleoceanography* **28**, 143–152, doi:10.1029/2012PA002311 (2013).
6. Montagna, P. *et al*. Li/Mg systematics in scleractinian corals: calibration of the thermometer. *Geochim. Cosmochim. Acta* **132**, 288–310, doi:10.1016/j.gca.2014.02.005 (2014).
7. Fowell, S. E. *et al*. Intrareef variations in Li/Mg and Sr/Ca sea surface temperature proxies in the Caribbean reef-building coral *Siderastrea siderea*. *Paleoceanography* **31**, 1315–1329, doi:10.1002/2016PA002968 (2016).
8. Ross, C. L., DeCarlo, T. M. & McCulloch, M. T. Calibration of Sr/Ca, Li/Mg and Sr-U paleothermometry in branching and foliose corals. *Paleoceanogr. Paleoclimatology* **34**, 1271–1291, doi: 10.1029/2018PA003426 (2019).
9. Cuny-Guirriec, K. *et al*. Coral Li/Mg thermometry: caveats and constraints. *Chem. Geol*. **523**, 162–178, doi:10.1016/j.chemgeo.2019.03.038 (2019).
10. Stewart, J. A. *et al*. in review
11. Hathorne, E. C. *et al*. Interlaboratory study for coral Sr/Ca and other element/Ca ratio measurements. *Geochemistry, Geophys. Geosystems* **14**, 3730–3750, doi:10.1002/ggge.20230 (2013).
12. Dickson, A. G. Thermodynamics of the dissociation of boric acid in synthetic seawater from 273.15 to 318.15 K. *Deep-Sea Res.* **37**, 755–766, doi:10.1016/0198-0149(90)90004-F (1990).
13. DeCarlo, T. M., Holcomb, M. & McCulloch, M. T. Reviews and syntheses: revisiting the boron systematics of aragonite and their application to coral calcification. *Biogeosciences* **15**, 2819–2834, doi:10.5194/bg-15-2819-2018 (2018).
